# Congenital thoracic malformations in pediatric patients: two decades of experience

**DOI:** 10.1590/S1806-37132015000004374

**Published:** 2015

**Authors:** Tatiane da Anunciação Ferreira, Isabella Santana Santos Chagas, Regina Terse Trindade Ramos, Edna Lúcia Souza

**Affiliations:** 1 Pediatric Pulmonologist. Department of Pediatric Pulmonology, Professor Edgard Santos University Hospital, Federal University of Bahia School of Medicine, Salvador, Brazil; 2 Pediatrician. Lagarto Regional Hospital, Federal University of Sergipe, Lagarto, Brazil; 3 Adjunct Professor. Department of Pediatrics; and Graduate Program in Medicine and Health, University of Bahia School of Medicine, Salvador, Brazil; 4 Associate Professor. Department of Pediatrics; and Coordinator, Department of Pediatric Pulmonology, Professor Edgard Santos University Hospital, Federal University of Bahia School of Medicine, Salvador, Brazil

## To the Editor:

Congenital thoracic malformations constitute a heterogeneous group of developmental disorders, involving lung parenchyma, arterial supply, and venous drainage.^(^ 1 ^)^ Their etiology is embryologic, and their clinical presentation and severity vary according to the degree of pulmonary involvement and their location in the thoracic cavity.^(^ 2 ^)^ They can be asymptomatic-their diagnosis being based on incidental chest X-ray findings-or cause early and severe respiratory symptoms. ^(^ 2 ^,^ 3 ^)^ Although prenatal ultrasound has allowed intrauterine diagnosis of congenital thoracic malformations,^(^ 1 ^,^ 4 ^)^ controversy remains regarding the nomenclature and treatment. Congenital thoracic malformations include pulmonary sequestration, congenital pulmonary airway malformations (formerly known as congenital cystic adenomatoid malformation), congenital lobar emphysema, bronchogenic cyst, congenital diaphragmatic hernia, diaphragmatic eventration, pulmonary vascular malformations, bronchial atresia, pulmonary hypoplasia, and pulmonary agenesis.^(^ 1 ^)^ Chest X-rays can show localized hypertranslucency, cystic images, decreased volume in the right or left hemithorax, changes in the pulmonary vasculature, and condensation images. The objective of this letter is to report the clinical data and therapeutic management of patients with congenital thoracic malformations followed in the Pediatric Pulmonology Department of the Federal University of Bahia University Hospital, in the city of Salvador, Brazil, between 1991 and 2013. 

This was a retrospective observational study (case series) of 26 patients with congenital thoracic malformations diagnosed radiologically, surgically, or both. Each medical chart was systematically analyzed. The study was approved by the local research ethics committee. 

All patients had undergone chest X-rays. The presence of congenital thoracic malformations was confirmed by chest CT scans, in 24 children, and by contrast-enhanced examination of the esophagus, stomach, and duodenum, in 2 children. The following malformations were found: congenital lobar emphysema, in 6; pulmonary agenesis, in 5; congenital pulmonary airway malformations, in 4; pulmonary hypoplasia, in 3; cystic disease, in 2; congenital diaphragmatic hernia, in 2; bronchogenic cyst, in 1; lobar agenesis, in 1; pulmonary vascular malformations, in 1; and diaphragmatic eventration, in 1. Pulmonary agenesis is very rare, having occurred in 5 patients; of those, 4 had right lung agenesis, which is consistent with the literature,^(^ 5 ^)^ with only 1 asymptomatic child. None of the patients had been diagnosed with pulmonary sequestration. 

Of the 26 patients studied, 14 (53.8%) were male. All of the patients with congenital lobar emphysema were male, which is consistent with the literature.^(^ 6 ^)^ Pulmonary agenesis and pulmonary hypoplasia were more common in female patients, which is inconsistent with the literature. Pulmonary agenesis was found in only 1 male patient. 

The mean age at diagnosis was 12.4 months (range: 0-120 months). Although prenatal diagnosis of congenital thoracic malformations has increased, only 3 patients had been diagnosed prenatally. This demonstrates the need for improving prenatal care. 

Most of the patients with congenital thoracic malformations in the present study were symptomatic; that is, 22 (84.6%) of the 26 patients studied had at least one symptom (dyspnea, wheezing, tachypnea, or cyanosis). Of the symptomatic patients, 21 (95.4%) were symptomatic in the first year of life. Recurrent pneumonia occurred in 6 children, being more common in those with cystic disease (found on imaging studies). 

Pulmonary agenesis (particularly right lung agenesis) can be associated with other congenital abnormalities.^(^ 5 ^)^ Of the 26 patients studied, 11 had other malformations, including facial dysmorphism, heart disease, clubfoot, bilateral deafness, umbilical hernia, and inguinal hernia. All of the patients with pulmonary agenesis had other malformations, one of the patients having multiple birth defects and an incidental diagnosis of congenital thoracic malformation at the age of 7 months. 

Abnormal development of the aortic arch during embryogenesis-which was not observed in the present study-results in pulmonary hypoplasia,^(^ 5 ^)^ which in turn is a consequence of congenital diaphragmatic hernia. One patient had decreased pulmonary vascularization. 

The only case of lobar agenesis involved the left upper lobe, with no other malformations. The patient was treated conservatively. 

Pulmonary vascular malformations are characterized by abnormal communication between the pulmonary artery and vein, leading to right-to-left shunt.^(^ 7 ^)^ The main symptoms are dyspnea, palpitation, fatigue, and epistaxis. ^(^ 8 ^)^ The patient who had pulmonary vascular malformations had recurrent pneumonia, which resolved after surgical treatment. 

Congenital diaphragmatic hernia is a developmental defect of the diaphragm that allows abdominal viscera to reach the thoracic cavity. It usually causes symptoms at birth. In the present study, the 2 patients with diaphragmatic hernia were asymptomatic-which is very rare-having been diagnosed at ages 3 and 10 years. Both were referred for surgical correction. 

Diaphragmatic eventration results from a congenital structural defect or phrenic nerve injury, which can be iatrogenic or due to traction during birth.^(^ 9 ^)^ Although diaphragmatic eventration is usually asymptomatic, it can cause dyspnea and respiratory infection in infants.^(^ 9 ^)^ The patient with diaphragmatic eventration presented with tachypnea, and the radiological findings were mistaken for pneumonia. The patient was asymptomatic after surgical correction. 

**Figure 1 -:**
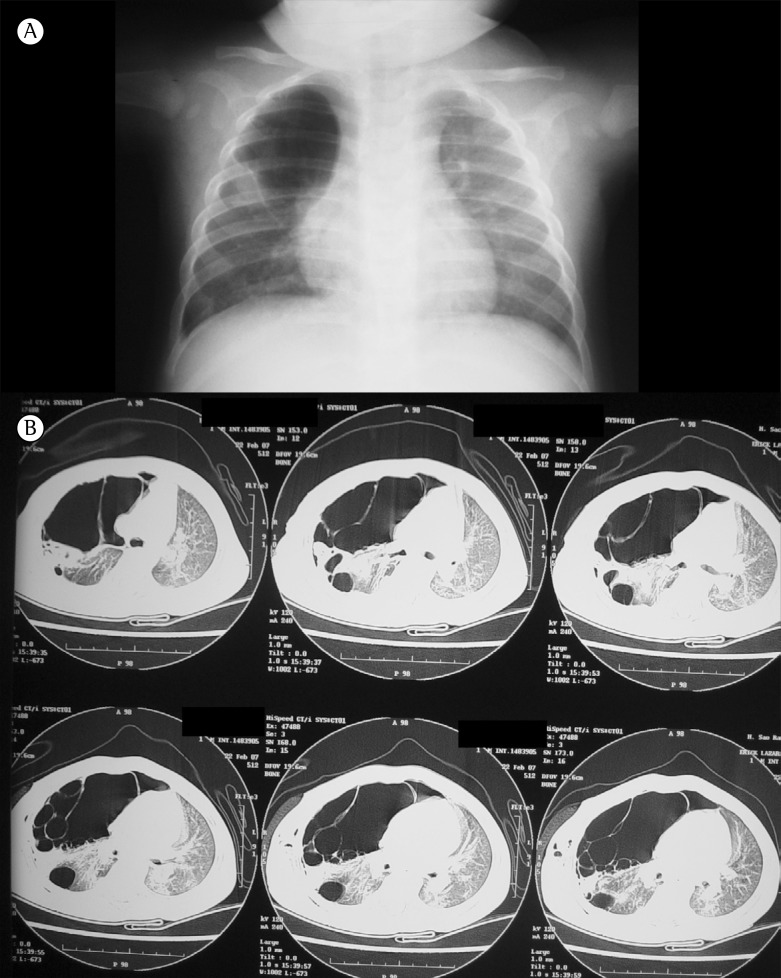
Chest X-ray and CT scans of a 5-month-old patient with respiratory distress. In A, chest X-ray showing hypertranslucency in the right upper lobe, without mediastinal shift. In B, chest CT scans showing cystic images of varying sizes in the right upper lobe. The patient underwent right upper lobectomy. Histopathological examination confirmed the presence of congenital pulmonary airway malformation.

Cystic disease is one of the most common congenital thoracic malformations.^(^ 2 ^)^ In the present study, it was observed in 7 patients, 5 of whom underwent surgery. Of those 5 patients, 4 had congenital pulmonary airway malformations and 1 had bronchogenic cyst. Although congenital pulmonary airway malformations are more common in boys,^(^ 2 ^)^ they were observed in two girls and two boys in the present study. After surgical treatment, 3 patients were classified as having type I malformation, i.e., large cysts (2-10 cm in diameter)-the most common type-and 1 was classified as having type II malformation, i.e., small cysts (0.5-2 cm in diameter). Figure 1 shows X-ray and CT findings of a child diagnosed with type I congenital airway malformations.

**Figure 2 -:**
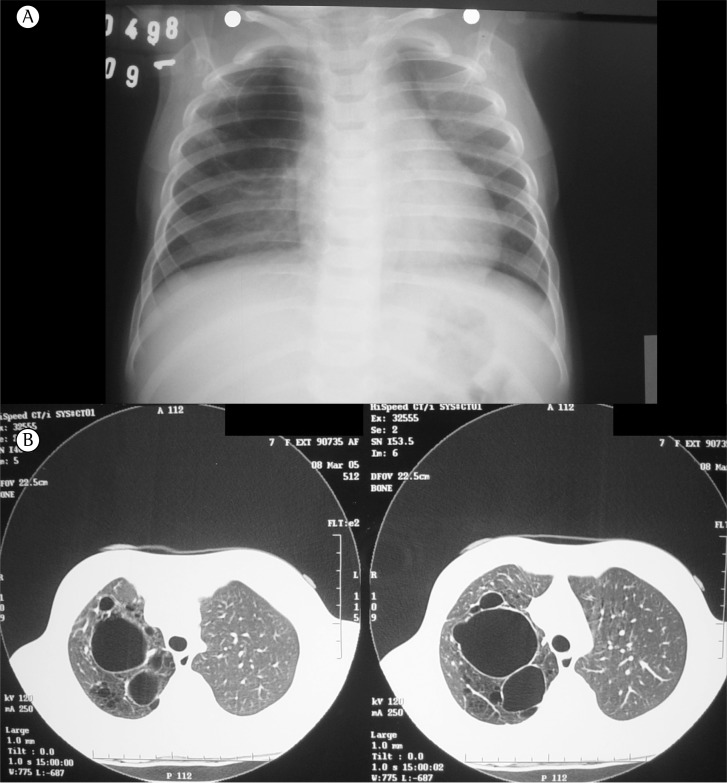
Chest X-ray and CT scans of a patient with a prenatal diagnosis of cystic lung disease. In A, posteroanterior chest X-ray (taken at age 6 months) showing cystic images in the right upper lobe. In B, chest CT scans showing cystic images of varying sizes in the right upper lobe. The patient underwent right upper lobectomy. Histopathological examination confirmed the presence of bronchogenic cyst.

Bronchogenic cyst is a benign, congenital mediastinal malformation whose natural course is undefined; malignant transformation can occur in adults.^(^ 10 ^)^ Although bronchogenic cyst is often asymptomatic, secondary infection and complications can occur.^(^ 10 ^)^ One of the patients with cystic disease had recurrent pneumonia; after surgery, the presence of bronchogenic cyst was confirmed (Figure 2). 

Congenital lobar emphysema results from bronchial or alveolar changes.^(^ 6 ^)^ Although lobectomy is indicated for symptomatic patients, there is uncertainty regarding the treatment of asymptomatic patients.^(^ 6 ^)^ In the present study, 4 patients with severe symptoms underwent lobectomy. All 4 had left upper lobe involvement, including the patient with bilateral disease (i.e., right and left upper lobe involvement). The two patients who were managed conservatively had right and left upper lobe lesions, respectively, and remained asymptomatic at follow-up. 

Most of the patients who underwent surgery progressed well. One patient had sepsis 48 h after having undergone lobectomy but responded favorably to antibiotic therapy. The only death was due to pneumonia, having occurred in the patient with pulmonary agenesis. 

There is no consensus regarding the treatment of congenital thoracic malformations.^(^ 1 ^)^ In some centers, surgical treatment is recommended for symptomatic patients only.^(^ 4 ^)^ In others, early surgical resection is recommended in all cases because of the risk of complications.^(^ 1 ^,^ 4 ^)^ Some authors recommend conservative treatment for asymptomatic patients because of the lack of data on long-term complications.^(^ 1 ^)^ In the present study, asymptomatic or mildly symptomatic patients were treated conservatively. 

Congenital lobar emphysema, pulmonary agenesis, and congenital pulmonary airway malformations were the most common congenital thoracic malformations in the study sample. Most of the patients had early symptoms, the most common clinical manifestations being respiratory distress and recurrent pneumonia. Regardless of the approach used, the prognosis was excellent. 
